# PATH-G: a proposed integrative motivational-cognitive-behavioral protocol for gambling disorder. A theoretically grounded 12-week workbook-based model with maintenance and relapse components

**DOI:** 10.3389/fpsyt.2026.1871785

**Published:** 2026-09-10

**Authors:** Pierluigi Diotaiuti, Marco Palombo, Stefania Mancone

**Affiliations:** Department of Human Sciences, Society and Health, University of Cassino and Southern Lazio, Cassino, Italy

**Keywords:** cognitive-behavioral therapy, gambling disorder, hierarchical protocol, integrative psychotherapy, intervention protocol, maintenance, motivational interviewing, PATH-G

## Abstract

Gambling Disorder (GD) is a multifactorial behavioral addiction characterized by persistent gambling despite significant harm, and typically involves cognitive distortions, urge dysregulation, shame, interpersonal strain, avoidant coping, and progressive erosion of self-trust. The present manuscript introduces PATH-G (Progressive Adaptive Treatment Hierarchy for Gambling Disorder), a proposed, theoretically grounded and not yet empirically evaluated, therapist-guided, workbook-assisted, 12-week psychotherapy protocol for adults with GD, followed by a structured maintenance phase and a dedicated relapse module. PATH-G is designed as a clinical pathway to be delivered within psychotherapy rather than as a stand-alone self-help package. A central premise of PATH-G is that motivation should not be treated as a static pretreatment characteristic, but as a fluctuating therapeutic target that requires repeated assessment and active clinical work across the entire pathway. Accordingly, PATH-G integrates motivational interviewing-informed strategies, stage-sensitive treatment planning, values clarification, collaborative goal revision, and relapse-sensitive recommitment procedures alongside cognitive-behavioral, experiential, and schema-informed techniques. PATH-G is intentionally hierarchical rather than loosely eclectic. Cognitive-behavioral therapy (CBT) and motivational work form the evidence-based core; mindfulness, grounding, and impulse-regulation methods function as adjunctive regulatory tools; and schema-informed, reflective, humanistic, and Gestalt-derived exercises are used to deepen formulation, identity reconstruction, and maintenance. The weekly sequence progresses from engagement, assessment, behavioral monitoring, and psychoeducation to trigger analysis, cognitive restructuring, impulse delay, emotional regulation, graded behavioral substitution, consolidation of abstinence, values-based future planning, maintenance, and relapse analysis. Distinctive features include the explicit division between in-session and between-session therapeutic tasks, collaborative treatment commitment and safety planning, self-monitoring forms, graded substitution of gambling time with ordinary-life activities, motivational anchors between sessions, mode-oriented maintenance exercises, and a non-punitive relapse framework. The main contribution of PATH-G is translational and clinical. It operationalizes a multimodal rationale into a progressive workbook that supports therapist guidance, patient participation, and continuity of care, while preserving case formulation, pacing decisions, and individual tailoring. Because PATH-G has not yet been empirically evaluated as a whole, it should be presented as a theoretically grounded intervention model rather than an outcome-validated stand-alone treatment. Future studies should test feasibility, acceptability, fidelity, and multidimensional outcomes of PATH-G in routine clinical settings.

## 1 Introduction

Gambling Disorder (GD) sits, in DSM-5, among the addictive disorders, defined by persistent and recurrent gambling behavior that produces clinically significant impairment or distress (1). Clinical descriptions of GD commonly extend beyond formal diagnostic criteria: patients bring distorted beliefs about chance and control, urges that escalate rapidly, and a degree of emotional dysregulation, comorbidity, and social and financial harm that goes beyond what “excessive gambling” suggests (2–5). Shame and concealment are frequently reported, together with demoralization and relational strain (6, 7). Qualitative and theoretical work also suggests that, for some patients, gambling may serve an emotion-regulatory or escape-based coping function rather than reward-seeking alone, including tension reduction, avoidance of aversive affect, or transient restoration of a sense of control; this function has been described in qualitative interview studies and is consistent with the emotionally vulnerable pathway identified in etiological models of problem gambling (8–10). This is treated as one plausible clinical function among others, not as a general or established mechanism, and is presented here as an interpretive frame consistent with these sources rather than as a demonstrated finding.

This clinical picture has direct implications for protocol design. Symptom suppression alone will not hold: the pathway has to work on behavior, cognition, affect regulation, meaning-making, and the maintenance of change over time, not just on the gambling episodes themselves. Equally important, many patients never reach treatment in the first place. Shame, stigma, and fear of disclosure keep people away, and the literature on help-seeking barriers is consistent on this point: self-stigma, a low perceived need for help, and a preference for handling the problem alone all delay or prevent engagement with services (6, 7, 11, 12). Current guidance goes further, linking these same barriers to gambling-related suicidality and warning that they keep people from even discussing their gambling with anyone (13). A protocol that ignores this reality, and simply assumes patients arrive ready and stay engaged, is not clinically useful. It has to be built for ambivalence, for fluctuating risk, and for continuity of care.

The present manuscript describes a 12-week operational workbook, built for use inside structured psychotherapy: one therapist-led session a week for three months, then a maintenance phase and a dedicated relapse module. It is not intended to substitute for the therapist. Its function is to extend the therapeutic work into the patient’s daily life, through structured forms, reflective tasks, behavioral assignments, motivational prompts, and relapse-prevention tools that live between sessions rather than during them. This approach is consistent with reviews of gambling interventions, which describe common practice as therapist-delivered work paired with structured self-help material, cognitive restructuring, coping skills training, relapse prevention, and motivational components (14). Between-session work is treated as a primary locus of change, while formulation, pacing, interpretation, containment, and risk management remain the responsibility of the therapist.

The manuscript’s contribution is not a new school of therapy; no such claim is made. Rather, it offers a working demonstration of how several therapeutic traditions can be organized into one coherent, progressive, operational pathway. In practical, operational terms, the protocol targets reduction of gambling behavior and associated harm, increased reflective self-monitoring, and strengthened emotional and motivational regulation; its later phases aim to support autonomy and a self-concept less organized around gambling, an outcome domain related to identity change constructs discussed in the addiction recovery literature (15, 16), though PATH-G does not yet report empirical data on this outcome and it should be regarded as a target for future assessment rather than a demonstrated effect.

## 2 Why an integrative, hierarchical protocol is clinically justified

The protocol is integrative, but it is not intended to be read as evidence-neutral eclecticism. Its components are organized hierarchically according to clinical function and relative evidentiary support. Meta-analytic and review evidence continues to support CBT as one of the most established psychological approaches for gambling-related harms. Recent syntheses indicate that psychological interventions are effective for GD and that face-to-face treatments show particularly strong effects, while umbrella review evidence supports CBT in reducing gambling severity, frequency, and expenditure (17–20). This evidence base has also crossed a formal threshold: the American Psychological Association’s Society of Clinical Psychology now lists CBT as an empirically supported treatment for gambling disorder. (20; see also Society of Clinical Psychology, n.d., https://div12.org/treatment/cognitive-behavioral-treatment-for-gambling-disorder) (21). This is taken as further support for positioning CBT at the core of the present protocol and as the benchmark against which any integrative addition must justify its own value.

Within the present protocol, CBT provides the primary architecture for identifying gambling-specific distortions, mapping reinforcement cycles, implementing stimulus control, analyzing antecedents and consequences, and restructuring beliefs such as illusion of control, gambler’s fallacy, and loss-chasing rationalizations.

Motivational work forms the second pillar of the core evidence-informed layer. This decision is not only theoretical but pragmatic. Treatment-seeking gamblers often present with ambivalence rather than stable commitment. Readiness to change has been shown to relate to gambling severity and reductions in gambling, while brief and combined motivational interventions have demonstrated clinical utility in several gambling studies and meta-analytic reviews (22–27). This evidence base should, however, be read alongside a more recent systematic review and meta-analysis, which found that no trial to date had established MI integrity and concluded that prior effect-size estimates may have overestimated the isolated effect of MI-informed interventions for problem gambling and gambling disorder (28); accordingly, the strength of this evidence is best characterized as provisional rather than firmly established, pending replication with integrity-verified trials.

The most closely related approach is Cognitive Motivational Behavior Therapy (CMBT; 29), which similarly integrates motivational interviewing into every phase of treatment rather than confining it to intake. The evidence supporting CMBT is promising, though it currently rests on a small number of trials: in a randomized trial against Gamblers Anonymous with 46 participants (30), CMBT reached a 94% probability of treatment completion, 95.7% of participants attended all 12 sessions, and 91.3% completed the 6-month follow-up, with lower gambling expenditure at follow-up than in the GA arm. Compared with standard CBT, CMBT performed comparably on gambling frequency, expenditure, motivation, and irrational cognitions, and showed an advantage specifically in session attendance and in money spent on gambling days in the year following treatment (31). PATH-G shares CMBT’s central premise that motivation should be assessed and addressed repeatedly throughout treatment rather than only at intake, while extending the pathway through three additional features, described below, that differentiate it structurally and functionally.

Recent guidelines recommend brief motivational interviewing to encourage further help-seeking in people reluctant to access services and emphasize therapeutic delivery that is empathic, non-stigmatizing, collaborative, and engagement-oriented (13). In this protocol, motivational work is therefore treated as a longitudinal mechanism of change rather than a preliminary intake procedure.

A second layer of the model includes adjunctive regulation strategies, especially mindfulness-informed exercises, grounding, urge delay, and breathing-based self-regulation. These tools are used to help patients tolerate urges and affective activation without immediate behavioral enactment. They are included as complementary methods rather than as stand-alone treatment models, consistent with the protocol’s practical aim of expanding coping repertoires in moments of high risk (32–39).

A third layer includes schema-informed, reflective, humanistic, and Gestalt-derived contributions. These elements are not presented as evidence-equivalent alternatives to CBT for GD. Rather, they are used to deepen case formulation and to support the later phases of treatment, especially when gambling serves functions linked to unmet emotional needs, punitive self-relations, chronic emptiness, or identity disturbance. Schema Therapy provides a language for maladaptive coping modes and for strengthening Healthy Adult functioning (40). Reflective and metacognitive exercises are used to increase awareness of internal states and reduce automatic enactment (41). Humanistic and Gestalt principles contribute authenticity, present-centered awareness, and embodied contact, all of which may be clinically useful when the patient must learn not only to stop gambling, but also to inhabit life without the symptom (42–44).

This hierarchy matters methodologically. It makes clear that the protocol is anchored first in interventions with stronger evidence for gambling-related harms, while using adjunctive and depth-oriented elements to address the broader clinical realities that often determine adherence, relapse vulnerability, and long-term recovery. In this sense, the protocol is integrative by design, but disciplined in rationale.

To make this hierarchy, and its evidentiary basis, explicit rather than implicit, **Table 1** maps each main component of PATH-G onto the type and strength of evidence supporting it in Gambling Disorder specifically, whether that evidence is direct or extrapolated from related clinical areas, and the rationale for its inclusion. This mapping is intended to help readers distinguish empirically supported core elements from adjunctive techniques and from more theoretical or exploratory components whose contribution remains to be tested.

**Table 1:** Evidence mapping for the main components of PATH-G, distinguishing direct gambling-disorder evidence from evidence extrapolated from related clinical areas.

PATH-G component	Type/strength of evidence in gambling disorder	Direct or extrapolated	Rationale for inclusion in PATH-G
Cognitive-behavioral therapy (restructuring, stimulus control, functional analysis)	Strong: consistent support across multiple meta-analyses and umbrella reviews (18, 20); recognized as an empirically supported treatment for GD by the APA Society of Clinical Psychology	Direct (GD-specific trials)	Core, evidence-based architecture of the protocol
Motivational interviewing-informed strategies	Moderate: earlier RCTs and meta-analyses reported gambling-specific effects (22, 26), but a more recent systematic review and meta-analysis found that no trial had established MI integrity and concluded that prior effect-size estimates may have overestimated the isolated effect of MI-informed interventions (28); this rating has been revised downward accordingly and should be considered provisional	Direct (GD-specific trials)	Addresses ambivalence and fluctuating motivation; second evidence-informed pillar
Mindfulness, grounding, and urge-delay techniques	Limited: we did not identify direct outcome trials in GD in this scan; stronger evidence base in substance use disorders and general emotion regulation (32, 36, 37)	Largely extrapolated, with some GD-specific studies	Adjunctive regulation tool for urge tolerance; theoretically plausible, not weighted as core evidence
Schema-informed work (maladaptive modes, Healthy Adult functioning)	Limited: we did not identify direct outcome trials in GD in this scan; evidence base established mainly in personality disorder and complex trauma populations (40)	Extrapolated	Deepens case formulation for presentations linked to unmet emotional needs; exploratory in this context
Reflective and metacognitive exercises	Limited: we did not identify direct GD outcome evidence in this scan; theoretical grounding in mentalization-based approaches (41)	Extrapolated	Supports awareness of internal states preceding gambling episodes; theoretical and exploratory
Humanistic and Gestalt-derived exercises	Limited: we did not identify controlled outcome evaluations in GD in this scan, and controlled outcome literature is limited even within their originating traditions (42, 44)	Extrapolated/theoretical	Adds experiential depth in later phases; explicitly exploratory and in need of evaluation
Non-punitive relapse-prevention framework	Moderate: established in the broader addiction relapse-prevention literature (45, 46); GD-specific outcome data more limited	Extrapolated, with partial direct support	Structures the maintenance and relapse modules
Workbook/between-session self-monitoring format	Mixed: stand-alone self-help shows weaker effects than therapist-guided, combined formats (14)	Direct, but favoring combined delivery over the format alone	Operationalizes between-session work; used only within therapist-guided delivery, never as stand-alone self-help

This table is intended to make explicit which components of PATH-G rest on direct empirical support in Gambling Disorder and which are included as theoretically or clinically plausible adjunctive elements pending future evaluation, consistent with the overall characterization of PATH-G as a proposed, not yet empirically validated, protocol.

The ratings in **Table 1** reflect a non-systematic scan of the gambling-disorder treatment literature conducted for this manuscript, rather than a formal systematic review, and should be read as indicative summaries rather than as output of a standardized grading framework (e.g., GRADE). “Strong” denotes consistent support across multiple meta-analyses or umbrella reviews; “Moderate” denotes support from randomized trials whose effect estimates are smaller, less consistent, or have been qualified by more recent systematic reviews; “Limited” denotes the absence of direct controlled trials in GD identified in this scan, with support drawn mainly from related clinical populations; “Mixed” denotes evidence pointing in different directions depending on delivery format. Statements that direct outcome trials were not identified describe the result of this literature scan rather than a categorical claim that no such trials exist.

As **Table 1** makes explicit, the evidence supporting individual components of PATH-G is not uniform. The third-layer components in particular (schema-informed, reflective, humanistic, and Gestalt-derived exercises) lack direct outcome trials in Gambling Disorder and, in some cases, have a limited controlled outcome literature even within their originating clinical traditions. We include them as theoretically and clinically plausible adjunctive elements, on the basis of case formulation needs commonly reported in this population, rather than as components with an established, direct evidence base. Their expected contribution to outcome should be regarded as a hypothesis requiring future empirical testing, not as a demonstrated effect, and future dismantling or component studies would be needed to establish whether they add incremental value over the CBT and motivational core.

The clinical rationale for several protocol components is also strengthened by neurobiological evidence, although a full review of this literature is beyond the scope of the present manuscript. Gambling disorder involves dysregulation of dopaminergic reward circuits (including the nucleus accumbens, prefrontal cortex, and ventral striatum) that produce elevated salience for gambling cues, reduced inhibitory control, and heightened sensitivity to near-miss events that maintain gambling persistence despite net losses (2, 4). This neurobiological substrate informs three specific protocol choices. First, the early introduction of psychoeducation on randomness and probability (Week 2) directly targets gambling-specific cognitive distortions; we hypothesize that these distortions are partly maintained by reward-circuit sensitization, though this specific link has not been directly tested in intervention research and is offered here as a plausible mechanism rather than an established one. Second, urge delay and mindfulness-based regulation techniques (Week 5) address the neurobiological urgency of craving at the level of behavioral response inhibition rather than only cognitive appraisal. Third, graded substitution and reward reallocation are hypothesized to function not only as behavioral alternatives but as partial substitutes for the dopaminergic reward gambling provides, potentially reducing the relative motivational salience of gambling cues over time; this is a theoretical proposal rather than a tested mechanism, and no direct neurobiological measure is included in the present protocol to evaluate it. These mechanistic connections are presented as plausible explanatory frameworks rather than demonstrated causal pathways; direct neurobiological outcome measures are not part of the proposed monitoring framework and should be considered a priority for future mechanistic research.

## 3 Motivational work as a cross-cutting mechanism of change

In the present model, motivation is not treated as a static pretreatment characteristic but as a fluctuating therapeutic target that requires ongoing assessment and intervention. This choice responds directly to the lived course of GD. A patient may enter treatment after acute losses or family pressure, show strong early determination, then become demoralized by urges, boredom, shame, or partial setbacks. Another patient may appear unmotivated at intake, yet become increasingly engaged once the function of gambling is named with accuracy and without blame. For this reason, ambivalence is conceptualized not as resistance in a pejorative sense, but as clinically meaningful material to be explored collaboratively.

Within CMBT, motivational enhancement is conceptualized primarily as a mechanism for improving retention and treatment adherence, a clinically critical objective, as approximately 40% of adults with gambling disorder will prematurely discontinue treatment, with most discontinuing immediately following the first session. The present protocol shares this retention-oriented rationale, but extends motivational work into two additional functional domains not foregrounded in CMBT: the motivational processing of identity transitions, specifically, the shift from crisis-driven stopping to values-based non-gambling identity, which is addressed explicitly in Weeks 11–12 and the maintenance phase; and the motivational repair function after lapses, which is operationalized through schema-informed mode analysis and a structured post-lapse recommitment procedure. These functions are not merely motivational maintenance; they address what the literature on recovery increasingly identifies as distinct clinical processes requiring specific intervention (47, 48).

The transtheoretical model is used as an orienting map, not as a rigid staging device. Motivational positioning is explored at intake, revisited at mid-treatment, and re-examined after lapses, fatigue, or major shifts in life context (49). The workbook’s initial scaling of importance, perceived usefulness, difficulty, and commitment is therefore clinically valuable, but insufficient on its own. PATH-G explicitly treats motivation as something that must be supported across transitions: entering treatment, reducing gambling exposure, moving toward abstinence, tolerating the emotional aftermath of non-gambling, and restoring commitment after lapses.

Several motivational tasks are embedded across the protocol. First, the patient is repeatedly asked to articulate reasons for change in relation to concrete life domains such as children, partnership, work, dignity, health, and freedom. Second, discrepancy is explored between what gambling promises and what it actually delivers, between the immediate appeal of gambling and its long-term costs, and between the patient’s preferred identity and the self-state enacted during gambling. Third, graded substitution tasks are framed not merely as behavioral prescriptions but as opportunities to experience competence, authorship, and visible benefit. Fourth, lapses are processed through recommitment rather than humiliation.

The therapist stance is central. Contemporary guidance recommends that interventions for gambling-related harms be delivered in ways that are understanding, empathic, supportive, helpful, ownership-promoting, non-stigmatizing, and dialogical, with continuity of care wherever possible (13). The protocol therefore assumes a motivational style in which the therapist asks, elicits, summarizes, reflects discrepancies, normalizes ambivalence, and helps the patient convert vague intention into operational goals. This stance is especially important in GD, where concealment, self-reproach, and fear of judgment are common barriers to sustained participation.

Motivational work is explicitly distributed across the full sequence:

- Weeks 1-2, motivation is assessed, named, and linked to initial goals, concerns, and willingness to engage in monitoring.
- Weeks 3-4, trigger analysis and cognitive restructuring are used to expose the internal logic by which gambling maintains itself despite stated intentions.
- Weeks 5-6, urge management and emotion regulation are reframed as experiences of self-efficacy rather than simply techniques.
- Weeks 7-10, motivational work focuses on fatigue, discouragement, ownership of abstinence, and redefinition of reward.
- Weeks 11-12 and maintenance, values clarification broadens motivation from crisis-driven stopping to life-direction.
- In the relapse module, motivation is repaired through a non-punitive, function-based analysis that protects the change process from collapse into all-or-nothing thinking.

The practical implication is that every major transition in the protocol has a motivational task. The patient is not merely asked to comply; the patient is helped to recover authorship.

## 4 Intervention architecture and treatment stance

For the purposes of clinical implementation and future research citation, the present protocol is referred to hereafter as PATH-G (Progressive Adaptive Treatment Hierarchy for Gambling disorder), a Hierarchical Integrative Workbook-Assisted Protocol for Gambling Disorder. The protocol is organized as a progressive 12-step path delivered across twelve weeks, followed by maintenance and relapse sections. Each week combines therapist-led work with structured between-session tasks. The design principle is cumulative: early steps privilege observation and engagement; intermediate steps focus on cognitive, behavioral, emotional, and motivational change; later steps consolidate abstinence, identity, and future direction.

A defining structural feature is the explicit distinction between what is done in session and what is done outside the consulting room. The workbook repeatedly communicates that everyday life is the field in which automatic patterns become visible and change is rehearsed. This division of labor supports accountability without collapsing treatment into homework compliance. The therapist uses between-session material to refine formulation, identify obstacles, reinforce progress, and adapt pacing.

At treatment entry, the patient completes a baseline questionnaire on gambling time, money spent, preferred gambling activities, subjective appeal of different gambling forms, current motivation, expected difficulty, and commitment to change. The revised protocol renames the original “honor agreement” as a Collaborative Treatment Commitment and Safety Plan. This shift in language matters. It preserves the clinical function of structure and accountability while avoiding moralizing connotations. The plan typically includes attendance, abstinence or reduction goals according to case formulation, financial protection, restriction of access to gambling opportunities, emergency contacts, therapist communication, and a safety framework for crises or relapse risk.

The therapeutic alliance is treated in this protocol not only as a relational backdrop but as an active clinical mechanism. It is one of the most consistently identified predictors of outcome across psychotherapy modalities (50), and readiness to change (49), irrational beliefs (2), and coping skills (17, 18) are widely discussed as potential clinical determinants of outcome in the gambling treatment literature more broadly, though this specific paragraph reflects a general clinical synthesis rather than findings from any single study. The Collaborative Treatment Commitment and Safety Plan is therefore not primarily a behavioral contract; it is an early alliance-building device that gives the patient’s stated reasons for change a formal, visible, jointly authored form. Its renegotiation at mid-treatment (Week 6) and after lapses serves the same alliance-protective function.

Repeated weekly elements include review of homework, emotional check-ins, self-ratings of time and loss experience, coping analysis, and focused technical work. This repetition is purposeful. It creates rhythm, allows small changes to become visible, and gives form to continuity. At the same time, the therapist should use the workbook flexibly. Patients with high shame, severe comorbidity, cognitive limitations, unstable housing, active substance use, or elevated suicide risk may require slower pacing, more motivational work, more external supports, or adaptation of literacy demands; this is consistent with evidence that gambling problems frequently co-occur with other psychiatric conditions and may require integrated or adapted care (3, 51).

The workbook in its standard form assumes a reading level broadly consistent with lower-secondary education and requires the capacity to complete written reflective tasks between sessions. Where literacy demands pose a barrier, therapists may adapt delivery through: oral completion of diary entries, with the therapist scribing during session; simplified visual formats for monitoring forms (e.g., emoji-based emotion rating scales, visual urge thermometers); audio recordings replacing written motivational anchors; and reduced between-session task volume with more explicit in-session processing of each completed element. Patients with mild cognitive impairment, limited formal education, or significant working memory difficulties may benefit from a slower pacing schedule (for example, extending the 12-week sequence to 16–20 weeks) with greater use of concrete behavioral tasks relative to reflective and identity-oriented exercises.

## 5 Week-by-week therapeutic sequence

PATH-G is delivered as one individual session per week for 12 consecutive weeks. Each session is designed for a standard 50-minute clinical hour, although sessions in Weeks 1 and 6 (which include comprehensive assessment, motivational review, and collaborative revision of the treatment plan) may extend to 75–90 minutes where service contexts permit. Maintenance sessions are recommended at monthly intervals for a minimum of three months following Week 12, with session length of approximately 50 minutes. The relapse module is activated as needed and typically requires two to three additional sessions.

The weekly sequence above can also be condensed by phase, as shown in **Table 2**. The phase logic is clinically useful because it highlights that the protocol moves from observation and engagement to self-regulation, then from regulation to identity and continuity. This temporal architecture is especially important in GD, where treatment can fail if it remains confined to early symptom reduction and does not help the person build a life structure capable of sustaining non-gambling over time.

**Table 2:** Protocol overview by phase.

Phase	Weeks	Primary clinical tasks	Motivational tasks
Assessment and engagement	1-2	Baseline assessment, emotional and behavioral monitoring, psychoeducation, treatment commitment and safety planning, early reduction of denial.	Importance and confidence scaling, eliciting reasons for change, discrepancy building, treatment engagement.
Functional analysis and cognitive work	3-4	ABC analysis, trigger mapping, schedule disruption, substitution of gambling time, identification and restructuring of gambling distortions.	Linking automatic thoughts to ambivalence, strengthening ownership of first behavioral changes.
Impulse and affect regulation	5-6	Urge delay, grounding, breathing, mindfulness, adaptive coping, reward budgeting, mid-treatment review.	Reframing self-regulation as self-efficacy; motivational renewal at the midpoint.
Consolidation and abstinence	7-10	Expansion of non-gambling days, mirror-based reflection, pattern interruption, relapse-prevention planning, maintaining behavior analysis.	Working through fatigue, protecting commitment, redefining reward and non-gambling identity.
Identity and future orientation	11-12	Values clarification, long-term goals, Healthy Adult consolidation, future planning, transition to maintenance.	Moving from crisis-driven stopping to values-based continuity.
Continuation of care	Post-12	Maintenance exercises, routine protection, daily mode check-ins, values-based living, relapse-sensitive case review.	Recommitment after setbacks, preserving authorship over time.

### 5.1 Week 1: assessment, engagement, and motivational positioning

The opening module establishes baseline behavior, current gambling patterns, emotional states before and after gambling, subjective appraisal of gambling time, and motivational positioning within the stages of change. The collaborative treatment commitment and safety plan is introduced. Clinically, this week serves three functions: data gathering, symbolic transition into treatment, and early activation of agency. The gambling diary begins immediately so that therapy starts from observation rather than vague recollection. Where services require standardized monitoring, the diary can be complemented by validated gambling-severity or treatment-outcome instruments such as the CPGI/PGSI or GAMTOMS (52, 53).

### 5.2 Week 2: psychoeducation and discrepancy building

The second week introduces psychoeducation on reinforcement, randomness, probability, the illusion of control, and the discrepancy between what gambling promises and what it actually delivers. Patients are invited to write or discuss the contrast between anticipated gain and actual consequence. Motivationally, this week strengthens discrepancy without confrontation. The goal is not to shame the patient into change but to make the maintenance cycle legible; this is especially relevant because near-misses, perceived personal control, and biased interpretations of randomness can maintain gambling persistence (2).

### 5.3 Week 3: trigger identification and initial behavioral substitution

The third week introduces ABC functional analysis and mapping of internal and external triggers, including boredom, stress, anger, loneliness, availability of money, social exposure, and online access. Patients are asked to alter their habitual gambling schedule and to renounce one usual gambling day in favor of planned alternative activities. Motivationally, this is the first week in which intention is translated into visible behavioral authorship.

### 5.4 Week 4: cognitive restructuring of gambling distortions

This module targets distorted beliefs such as loss chasing, gambler’s fallacy, magical luck-based thinking, and dichotomous expectations about winning, recovery, or self-control. Through Socratic discussion and written alternatives, patients begin to replace gambling-justifying thoughts with more reality-based formulations. Cognitive work is linked to motivational work by emphasizing that distorted thoughts often speak in the voice of immediate urge rather than long-term intention.

### 5.5 Week 5: impulse management and reward reallocation

Week 5 focuses on delaying the urge, healthy distraction, grounding, breathing, and mindfulness-based urge tolerance. The patient also begins to convert avoided gambling into measurable savings and to invest those savings in concrete rewards or meaningful goals. This is both a behavioral and motivational turning point: the patient is helped to experience that non-gambling is not only restraint, but also visible gain. Mindfulness-informed craving and urge-management strategies are best understood here as adjunctive skills that may reduce craving intensity and improve distress tolerance, rather than as a replacement for CBT or motivational work (36, 37, 39).

### 5.6 Week 6: mid-treatment motivational renewal and emotion regulation

At the midpoint, the workbook revisits importance, difficulty, commitment, and the reasons for change. This timing is clinically important because initial effort may be followed by fatigue, irritability, or emotional backlash. The technical focus shifts to affect tolerance, adaptive coping, and the use of emotional regulation strategies without gambling. The therapist explicitly reviews ambivalence and renews commitment in a collaborative manner.

### 5.7 Week 7: strengthening abstinence and pattern interruption

The seventh week marks the transition from reduction to stronger abstinence-oriented work when clinically indicated. The patient continues functional analyses, expands non-gambling days, and identifies maintaining behaviors that keep gambling psychologically available even when gambling frequency has decreased. Motivational work now targets the difference between stopping a behavior and relinquishing an identity-linked ritual.

### 5.8 Week 8: reflective self-observation and disillusionment of the gambling setting

Reflective tasks become more explicit. Patients observe gambling environments and other gamblers before participating, noting posture, facial expression, hand movement, atmosphere, odor, noise, crowding, and emotional tone. These observational exercises weaken fusion with the gambling scene by converting seduction into description. Mirror-based reflective work may also be introduced to strengthen self-recognition and reduce dissociative momentum. This task functions, in effect, as a graded, therapist-supervised cue-exposure procedure: gambling environments and related stimuli are approached deliberately, under controlled conditions, rather than avoided, with the aim of reducing cue-reactivity and craving through repeated non-reinforced exposure (54). A systematic review and meta-analysis of exposure-based interventions for GD found large reductions in gambling craving at post-treatment and follow-up, alongside reductions in gambling severity and erroneous beliefs, though the authors note a high risk of bias across the small number of available trials and call for larger controlled studies (55); the exercise is therefore best regarded as a promising but not yet well-established technique. This exercise requires careful clinical judgment regarding patient selection. It is contraindicated for patients with marked urge reactivity, those in early treatment phases, patients whose gambling is primarily online (where environmental exposure is ambient rather than location-specific), and patients with active comorbid substance use. The therapist should assess urge intensity, perceived control, and current motivational stability before assigning this task, and should have a clear safety plan in place if the patient reports difficulty disengaging from the observed environment. Before the task, the therapist records baseline craving on a 0–10 subjective urge scale; the same scale is re-administered immediately after the exercise and again at the start of the following session, so that any increase in craving or gambling risk is identified and tracked rather than assumed to resolve on its own. If craving rises substantially after the exercise, the therapist uses the same in-session regulation strategies introduced in Week 5 (urge delay, grounding, breathing) before ending the session and schedules a check-in contact within 48 hours; a marked or sustained increase in craving, or any gambling episode following the exercise, should lead the therapist to discontinue this exercise for that patient and shift to the guided-imagery alternative described below. Where the exercise is deemed too high-risk, a guided imagery version, imagining the environment in session with therapist support, may serve a similar defusion function with lower exposure risk.

### 5.9 Week 9: coping styles, schemas, and emotional meaning

By this point, the patient has enough material to examine recurrent coping patterns and the emotional needs gambling may have been regulating. Schema-informed work helps distinguish the impulsive or avoidant mode from the broader self and introduces the possibility of a more protective and regulating Healthy Adult stance. Motivational work is deepened by linking change not only to consequences but also to unmet needs that deserve safer and more durable responses.

### 5.10 Week 10: relapse-prevention planning in advance

The tenth week formalizes relapse-prevention planning before a major lapse occurs. High-risk situations, warning signs, justifying thoughts, and emergency responses are mapped in writing. This anticipatory work is framed as competence rather than pessimism. The therapist reinforces the idea that protecting change is part of treatment maturity, not evidence of weak motivation. This framing is consistent with relapse-prevention models in addiction treatment, which conceptualize lapse analysis, high-risk situations, coping responses, and the abstinence violation effect as central treatment targets (45, 56, 57).

### 5.11 Week 11: values clarification and future-direction planning

The protocol broadens from symptom control to life direction. Patients identify values such as dignity, responsibility, authenticity, trust, perseverance, serenity, gratitude, or freedom, and translate them into small daily acts. Long-term goals begin to be organized across work, health, relationships, growth, and well-being. Motivation is now reframed from fear-based abstinence to values-based continuity.

### 5.12 Week 12: consolidation of non-gambling identity and transition to maintenance

The final week consolidates gains, reviews the entire sequence, identifies the most useful tools, and prepares for the maintenance phase. The patient is encouraged to describe what has changed in behavior, emotion, time use, self-perception, and future orientation. The clinical objective is not triumphal closure but a realistic and durable transition from intensive phase to continued care.

The week-by-week description above provides the clinical rationale and content for each module. To support replication and clinical implementation, **Appendix A** provides illustrative session outlines for three representative PATH-G sessions: Session 1 (engagement, baseline assessment, and collaborative treatment commitment), Session 5 (urge delay and emotional regulation), and Session 11 (values clarification and identity consolidation). Each outline specifies phase content, approximate time allocation, and the key therapist stance appropriate to that phase of treatment. These outlines are not prescriptive scripts, pacing and emphasis remain the therapist’s clinical responsibility, but they operationalize the session structure in a format directly usable for training, supervision, and fidelity monitoring.

## 6 Core therapeutic devices

What distinguishes PATH-G from a collection of evidence-based techniques is not its components in isolation, but the way those components are made to work on each other. Several recurring devices give the protocol its operational character, and they are worth describing not merely as a list of features but as a clinical system.

The workbook’s most fundamental device is self-monitoring. Patients record gambling episodes, money spent or lost, urges, emotional states before and after, situational triggers, and their own appraisals of time use. This is not merely data collection. Recording externalizes behavior that typically unfolds in automatic, concealed, or dissociated states; it introduces a small but meaningful gap between impulse and enactment; and it gives the therapist concrete material for formulation work that cannot easily be fabricated or rationalized away. When a patient notes that three out of four episodes over the previous fortnight followed late-night online access after arguments, that is clinical information of a different order than self-reported impressions.

Graded substitution builds directly on what monitoring reveals. Across the weeks, patients commit to replacing increasing numbers of gambling occasions with ordinary activities, such as work, exercise, family time, rest, errands, social contact. The gradual logic matters. Recovery framed as the removal of gambling in a vacuum tends to produce demoralization and dropout; recovery framed as the progressive reconstruction of a livable daily ecology gives the patient something to move toward rather than only something to suppress. The reinforcement is visible, cumulative, and anchored in the patient’s own weekly record.

A structurally different kind of device appears in Week 8, where patients are asked to observe gambling environments rather than enter them. Instead of being absorbed into the scene (the lights, the pace, the social rituals of the gambling floor), the patient is positioned as a witness: noticing atmosphere, bodily tension, the expressions on other gamblers’ faces, the cost written into posture and gesture. This shift from participant to observer functions as an *in vivo* defusion exercise. Clinically, it is best understood as a graded cue-exposure procedure (Section 5), and is offered with the corresponding contraindications and craving-monitoring safeguards described there. Cognitive restructuring asks what the patient believes about gambling; the observational task asks what they notice when they encounter it without automatic behavioral absorption. For patients who have long experienced gambling environments as seductive and welcoming, phenomenological description can do what rational counter-argument alone cannot: it breaks the spell.

Between-session motivational anchors serve a different function. Slogans, reflective prompts, brief audio or video materials, reminders of reasons for change, these are not interventions with independent therapeutic status. They are continuity devices. Their clinical function is to prevent the motivational tone of the session from fully dissipating in the interval before the next one, particularly during high-risk moments in daily life.

Reward budgeting converts what the patient has not done (not gambled, not lost) into something concretely visible. Avoided gambling becomes documented savings; savings become reinvestment in specific life areas. This is less a financial exercise than a motivational one: it makes abstinence legible as gain rather than only deprivation, and it strengthens the reinforcement structure that sustains non-gambling over time.

In the later modules, the devices shift register. Mirror-based self-observation, values clarification, dialogue between the Healthy Adult and the Impulsive Child schema mode, the function-of-gambling diary, phenomenological exercises such as dialoguing with absence, these tools address a question that the earlier behavioral and cognitive work does not: not “How do I stop?” but “How do I inhabit a life that no longer needs gambling as its organizing ritual?” By the time a patient reaches Week 10 or 11, the central clinical problem has often shifted from urge management to the experiential vacuum that non-gambling leaves behind. The later devices are designed for precisely that phase of recovery.

The therapeutic devices described above are operationalized in the PATH-G workbook through structured forms and reflective prompts designed for between-session use. **Appendix B** provides three key exercises in their operational format: the Weekly Gambling and Urge Diary (Weeks 1–8), which externalizes the gambling cycle and provides the therapist with concrete formulation material; the Graded Substitution Planner (Weeks 2–10), which converts non-gambling commitments into a written, reviewable schedule; and the Post-Lapse Recovery Plan (Relapse Module), which structures the patient’s self-directed response within 24–48 hours of a lapse. All exercises are introduced and explained by the therapist before being assigned; none are designed for unsupported self-administration. Adaptations for low literacy and online gambling presentations are noted within the appendix.

## 7 Maintenance as continuation of care

The protocol treats maintenance as a core structural component rather than a mere appendix to the acute treatment phase. After the initial 12 weeks, the workbook continues with structured exercises aimed at protecting routines, reviewing initial goals, maintaining financial safeguards, strengthening a non-gambling identity, and preventing the narrowing of recovery to simple avoidance.

The maintenance logic is both behavioral and existential. On the behavioral side, the patient continues with routine protection, weekly or periodic diaries, review of high-risk contexts, and structured alternative activities. On the reflective side, the materials introduce monthly schema reviews, daily mode check-ins, reflection on the function gambling once served, conscious tolerance of boredom, brief dialogues between the Healthy Adult and the Impulsive Child, clarification of lived values, and long-term planning in areas such as work, health, relationships, organization, and personal development.

This is clinically important because recent literature increasingly argues that recovery in GD should not be reduced to abstinence alone; it is a multidimensional process involving harm reduction, functioning, quality of life, self-direction, and personally meaningful change (47, 48). The workbook’s values section is especially consistent with this broader perspective. Patients are invited to select values such as authenticity, integrity, dignity, responsibility, empathy, trust, perseverance, serenity, gratitude, and meaning, and to translate them into concrete present-tense acts. The framing is deliberate: not “Who do I abstractly wish to become?” but “How do I live this value today?”

The protocol also includes phenomenological exercises that are unusual in gambling manuals but clinically coherent in later recovery. Examples include the diary of emptiness as space, the exercise of chosen presence, dialogue with absence, and ritual-of-continuity practices. These tasks are not presented as replacements for evidence-based methods. Rather, they help the patient tolerate the experiential void that often emerges when gambling is no longer available as an organizing activity. In this sense, maintenance is understood not as white-knuckled resistance, but as the progressive construction of a life in which gambling becomes less necessary, less believable, and less identity-congruent.

One dimension systematically absent from the present protocol in its current form is the relational and family context. Gambling disorder consistently produces significant harm to partners, children, and other family members, and recent guidance explicitly recommends that services consider providing support not only to the person who gambles but also to those affected by their gambling (13). The involvement of Concerned Significant Others (CSOs) in treatment, whether through psychoeducation sessions, joint safety planning, or coordinated communication, has been associated with improved treatment engagement and reduced concealment. Interventions involving CSOs or Affected Others may approach gambling harms support holistically, offering the opportunity to reflect both the wider social and political factors which can cause and influence harm and to centre individual lived experience. The present protocol does not include a formal CSO component, and this is acknowledged as a structural limitation. A future version of the workbook should develop a parallel track for significant others, including psychoeducation on gambling disorder, guidance on non-enabling responses, and structured support for managing the emotional aftermath of financial harm and concealment, that can be delivered alongside the patient’s individual treatment pathway.

## 8 Relapse management as clinically informative repair

The relapse module is a core structural component of the protocol. Rather than presenting relapse as moral failure, the workbook distinguishes between a lapse and a full relapse, invites functional analysis of the episode, and directly targets the abstinence violation effect, the belief that one mistake invalidates all previous gains. This position is consistent with CBT-based relapse prevention and with contemporary guidance that emphasizes hope, continuity, and non-stigmatizing care (45, 56, 57).

Relapse analysis is organized around ABC reconstruction. Emotional antecedents such as boredom, anger, shame, loneliness, or emptiness are considered alongside situational antecedents such as access to money, online exposure, interpersonal conflict, or disruption of routine. The behavior itself is then reconstructed in sequence, and the consequences are analyzed across immediate relief, emotional aftermath, financial loss, concealment, and motivational collapse. Thoughts such as “I deserve this”, “just this once”, “I have already ruined everything”, or “I can recover if I continue” are brought into cognitive analysis and reformulated with therapist support.

Schema-informed interpretation deepens this work. A lapse may be understood as the temporary dominance of the Vulnerable Child, Impulsive Child, Detached Protector, or Punitive Critic, with insufficient support from the Healthy Adult. This framing reduces shame while increasing precision. The therapist can help the patient ask not only why the lapse occurred, but which emotional mode became dominant, what support was missing, and what would have strengthened regulation in that specific moment.

A particularly useful addition is a post-lapse recovery plan for the next 24-48 hours. The patient identifies whom to contact, what environment is safest, which immediate actions reduce escalation risk, how to protect money or access, and what statement best restores commitment without self-humiliation. In motivational terms, relapse work is framed as repair, not expulsion from treatment. The goal is to shorten the interval between lapse and re-stabilization.

## 9 Assessment, monitoring, and candidate implementation outcomes

For publication as a protocol paper, it is important to specify how the intervention could be monitored in routine practice and in future feasibility studies. The workbook already contains rich idiographic monitoring through diaries, emotional tracking, coping checklists, and functional analyses. This section proposes a complementary framework of process and outcome domains. In implementation terms, feasibility, acceptability, appropriateness, fidelity, adoption, and sustainability should be treated as distinct constructs rather than inferred only from symptom change (58).

The selection of change techniques embedded in the core and second-layer components of this protocol overlaps with several of the 19 techniques rated by clinicians in a recent Delphi consensus study (59), including motivational enhancement, cognitive restructuring of gambling distortions, and relapse prevention planning. Emotion regulation strategies were not among the techniques for which that study reported consensus, and schema-informed and identity-reconstruction techniques were not among the 19 techniques it evaluated; they therefore cannot be described as having received lower consensus ratings, and their inclusion in the third layer of PATH-G reflects the case-formulation rationale set out in Section 2 rather than a comparison with Delphi findings. Keshani et al. (59) themselves note that clinician consensus is not a proxy for treatment effectiveness and requires experimental corroboration; the overlap noted here is therefore descriptive and should not be read as external validation of the protocol’s hierarchical structure. It should also be noted that Keshani et al. (59) did not examine therapeutic alliance, readiness to change, irrational beliefs, or coping skills as mediators or determinants of treatment outcome; that study is a Delphi consensus exercise on clinician-rated technique relevance, not an outcome or mediation study, and should not be cited in support of claims about which clinical factors predict or drive change in gambling treatment.

Outcome monitoring should extend beyond simple abstinence. The literature on treatment outcomes and recovery in GD supports multidimensional assessment, including gambling behavior, severity, urges, harm, functioning, and broader recovery indicators (47, 48). Accordingly, the protocol is well suited to monitoring the following domains: gambling frequency; money spent or lost; gambling severity; urge intensity; emotional regulation; perceived self-efficacy to resist gambling; treatment attendance; between-session task completion; lapse-to-recovery interval; and changes in personally meaningful life areas such as work, health, relationships, and daily structure. Standardized gambling-specific instruments may be used alongside idiographic workbook data when service or research contexts require comparability (52, 53).

Process monitoring is equally important. Because motivation is treated as a cross-cutting target, the protocol should monitor readiness, confidence, ambivalence, and ownership of goals at multiple points rather than only at intake. The same applies to alliance and engagement. Recent guidelines also recommend routine collection of baseline data and treatment outcomes in gambling services, which strengthens the rationale for explicit monitoring plans in protocol development (13).

The protocol is also suitable for future implementation research focusing on feasibility, acceptability, fidelity, therapist usability, patient burden, and dropout. This matters because psychological treatments for gambling, while effective overall, are also vulnerable to attrition, and dropout patterns remain a clinically important concern (60). A protocol that relies on structured between-session work should therefore be evaluated not only for symptomatic change, but also for whether patients can realistically engage with its demands in routine care. These implementation outcomes should be defined *a priori* so that clinical effectiveness is not confused with feasibility of delivery (58).

Across the dimensions listed above, structured monitoring serves two distinct purposes in PATH-G: it provides the therapist with clinically actionable data at key decision points (intake, mid-treatment, post-lapse, and maintenance review), and it establishes the outcome infrastructure required for future feasibility and effectiveness research. **Table 3** summarizes the suggested monitoring domains, their clinical rationale, and illustrative indicators for each. Domains are organized to reflect the full breadth of PATH-G’s targets: from core symptomatic change (gambling behavior and severity) through process variables (motivational fluctuation, engagement, urge management) to recovery-oriented outcomes (functioning, values-based action, self-trust) that extend beyond abstinence. Not all domains require formal psychometric instruments in routine care; structured clinical ratings, session-level scaling, and workbook-based self-monitoring can provide a pragmatic, lower-resource alternative, although their measurement properties have not been formally evaluated against validated psychometric instruments, and any such substitution should be treated as a practical compromise rather than a demonstrated equivalence.

**Table 3:** Suggested monitoring domains for routine care and future feasibility studies.

Monitoring domain	Clinical rationale	Examples of indicators
Gambling behavior	Core symptomatic change	Days gambled; frequency of episodes; time spent gambling; money spent/lost.
Gambling severity	Tracks overall clinical burden	Clinical severity ratings; symptom count or validated severity measure.
Urges and craving	Captures proximal relapse risk	Self-rated urge intensity; frequency of high-risk urges; delay success.
Emotional regulation	Links gambling to affective coping	Use of adaptive coping strategies; emotional tolerance during high-risk periods.
Motivational process	Motivation is a fluctuating target, not only an intake variable	Importance/confidence ratings; reasons for change; change plan revision.
Engagement and adherence	Assesses feasibility of workbook-based care	Attendance; homework completion; quality of diary use; session continuity.
Relapse recovery	Measures resilience after setbacks	Lapse-to-recovery interval; use of safety plan; time to therapist contact.
Recovery and functioning	Extends outcomes beyond abstinence	Work, health, relationships, daily structure, values-based actions, self-trust.

To place the present protocol against the frameworks it most overlaps with, **Table 4** lines up standard CBT for gambling disorder, CMBT (29–31), brief Motivational Interviewing, and PATH-G across five dimensions, intervention architecture, motivational integration, maintenance and relapse specification, third-layer components, and evidence base. The table is not intended to rank these approaches; CMBT and standard CBT rest on a considerably stronger evidence base than the present proposal at this stage. Its purpose is to make the overlaps and structural differences already discussed above visible at a glance.

**Table 4:** Structural comparison of treatment frameworks for gambling disorder.

Dimension	Standard CBT	CMBT (29–31)	Brief MI	Present protocol
Duration	8–20 sessions (variable)	12 sessions (fixed)	1–4 sessions	12 weeks + maintenance + relapse module
Motivational integration	Pretreatment or minimal	Longitudinal adaptive (core mechanism)	Primary focus; time-limited	Longitudinal + identity reconstruction phase
Workbook/between-session structure	Variable; not a defined primary tool	Not formally documented	Not applicable	Yes. Central therapeutic arena with defined weekly tasks
Maintenance phase (formalised)	Rarely explicit	Not formally documented	No	Yes. Structured module with identity, values, and routine components
Relapse module (dedicated)	Partial (relapse prevention embedded)	Not formally documented	No	Yes. Schema-mode analysis, post-lapse 24–48h plan, AVE processing
Schema-informed/third-layer components	No	No	No	Yes. Third layer (adjunctive; lower evidentiary weight)
Gestalt/phenomenological techniques	No	No	No	Yes. Week 8 observational exercise; mirror-based reflection
Identity reconstruction exercises	No	No	No	Yes. Maintenance phase (dialogue with absence; diary of emptiness)
Empirical evidence base (GD-specific)	High: consistent support across multiple meta-analyses and umbrella reviews, and listed as an empirically supported treatment for GD (18–20)	Moderate-to-high: consistent positive findings across the two available GD-specific RCTs, but not yet meta-analyzed and based on a small combined sample (29, 30; RCT N ≈ 46); promising but not yet meeting the “High” bar defined above, which requires convergent meta-analytic or umbrella-review support	Moderate, recently revised downward: a 2025 systematic review and meta-analysis found no MI-integrity-verified trials in GD and concluded that prior effect estimates may have been overestimated (28)	None. Theory-informed; awaits feasibility testing
Fidelity framework specified	Variable across manuals	Partially (adherence to MI + CBT components)	MITI available for MI	Proposed YACS (61)-adapted checklist; see **Table 5**)
Target population	Adults with GD; some gender adaptations	Adults with GD; low-readiness subgroup	Adults pre-contemplation/contemplation	Adults with GD; gender and online gambling adaptations recommended

Structural comparison of the present protocol with existing evidence-based frameworks for gambling disorder. CBT = Cognitive Behavioral Therapy; CMBT = Cognitive Motivational Behavior Therapy; MI = Motivational Interviewing; GD = Gambling Disorder; EST = Empirically Supported Treatment; AVE = Abstinence Violation Effect; YACS = Yale Adherence and Competence System; MITI = Motivational Interviewing Treatment Integrity scale. Empirical evidence ratings refer to gambling disorder-specific controlled studies and follow the same non-systematic scan and rating logic described for **Table 1**: “High” denotes consistent support across multiple meta-analyses or umbrella reviews; “Moderate” denotes support from randomized trials whose effect estimates are smaller, less consistent, or qualified by more recent systematic reviews; “None” denotes the absence of direct outcome trials identified in this scan. The present protocol has no independent empirical evidence base; its comparative positioning is theoretical.

## 10 Fidelity assessment framework

For the protocol to be testable in future research, a minimum fidelity framework must be specified in advance. Treatment fidelity has two distinct dimensions: adherence (whether the prescribed techniques were delivered) and competence (how well they were delivered). The Yale Adherence and Competence System (YACS) (61) provides a reliable and valid model for assessing fidelity of psychosocial treatments, distinguishing components that are unique and essential, essential but not unique, and antithetical to the approach, rated on 7-point Likert scales by independent raters. Adapting this framework to the present protocol, a minimum fidelity checklist should include: (a) unique and essential components (such as week-by-week workbook delivery, explicit motivational review at Weeks 1, 6, and the relapse module, collaborative treatment commitment and safety plan, and graded substitution tasks); (b) essential but not unique components, such as cognitive restructuring, functional analysis, urge delay techniques, and relapse prevention planning; (c) antithetical components, as confrontational stance toward ambivalence, punitive framing of lapses, omission of maintenance phase, delivery of workbook as unsupported self-help without therapist oversight. Future feasibility studies should include at least 20% session audio or video recording rated by trained independent coders, with inter-rater reliability established before outcome analyses.

To make this framework directly usable in future research, adherence and competence should be operationalized with concrete scoring procedures rather than left at the level of general principle. Adherence is scored using the complete core-task checklist in **Table 5**, which specifies, for every phase of the protocol, the discrete items a rater checks against session recordings or session notes. Each item is scored 0 (not addressed), 1 (addressed but incomplete or superficial — for example, a form introduced but not completed collaboratively, or a technique named but not rehearsed with the patient), or 2 (fully delivered as specified — completed collaboratively, with explicit rationale given to the patient). A session-level adherence percentage is calculated as the sum of item scores actually achieved divided by the maximum possible score for the items applicable to that session (right-hand column of **Table 5**), multiplied by 100; a protocol-level adherence index is then calculated as the mean of session-level percentages across all sessions rated for a given case. The provisional 80% threshold proposed here refers to this protocol-level index, not to any single session, since occasional lower-scoring sessions (for example, sessions interrupted by an acute crisis) should not by themselves indicate inadequate overall delivery; whether this threshold is appropriate, or should instead be phase-specific, is an empirical question for future feasibility studies rather than a fixed criterion.

**Table 5:** Complete core-task adherence checklist by protocol phase, for use with the session-level and protocol-level adherence scoring procedure described in Section 10.

Phase (weeks)	Core adherence items (score 0–2 each)	Max score
Assessment and engagement (1–2)	Baseline assessment completed; self-monitoring form introduced and first entry completed collaboratively; Collaborative Treatment Commitment and Safety Plan drafted and signed; importance/confidence scaling administered.	8
Functional analysis and cognitive work (3–4)	ABC analysis conducted on at least one recent episode; individualized trigger map updated; at least one gambling-specific cognitive distortion identified and addressed with restructuring technique; graded substitution task assigned.	8
Impulse and affect regulation (5–6)	Urge-delay or grounding technique introduced and rehearsed in session; mid-treatment motivational review (importance/confidence re-scaled) completed; Collaborative Treatment Commitment reviewed and, if needed, renegotiated.	6
Consolidation and abstinence (7–10)	Non-gambling day expansion reviewed against target; relapse-prevention planning content covered; between-session task reviewed at session opening; behavior-analysis data (diary/self-monitoring) reviewed collaboratively.	8
Identity and future orientation (11–12)	Values-clarification exercise completed; long-term goals articulated in patient’s own words; transition-to-maintenance plan drafted.	6
Maintenance/continuation of care (post-12)	Maintenance check-in completed at the scheduled interval; PPPR (Personal Relapse Prevention Plan) reviewed and updated if indicated; mode/identity check-in completed.	6
Relapse module (as activated)	Lapse or relapse explicitly named and distinguished from treatment failure; functional analysis of the relapse episode completed; Collaborative Treatment Commitment and safety plan revisited; non-punitive framing maintained throughout (rated under competence, not adherence).	6

Each item is scored 0 (not addressed), 1 (addressed but incomplete or superficial — e.g., introduced but not completed collaboratively, or completed without explicit rationale given to the patient), or 2 (fully delivered as specified, with explicit collaborative completion and rationale). The relapse module is activated only when clinically indicated and is scored separately from the phase in which it occurs.

**Table 5** provides the complete adherence checklist referenced above, organized by protocol phase rather than by individual week, to keep the instrument usable without requiring twelve separate week-by-week forms.

Competence should similarly be defined with explicit behavioral anchors rather than a single unanchored 1–7 scale. For each of the four relational indicators described above (reflective listening relative to closed questioning, avoidance of confrontational language, collaborative goal-setting, and non-punitive response to ambivalence or lapses), raters assign a global score from 1 to 7, anchored as follows: 1–2 (low) indicates the indicator is largely absent or the therapist’s behavior contradicts it (e.g., confrontational or punitive language predominates); 3–4 (moderate) indicates inconsistent presence, observable in some but not most relevant moments; 5–6 (good) indicates consistent presence across the session; 7 (excellent) indicates consistent and skillful presence, including in difficult moments (e.g., maintaining a non-punitive stance immediately after a disclosed lapse). This anchoring follows the global-rating format used in MITI 4 (62). Both adherence and competence should be rated by at least two independent, trained raters on a minimum of 20% of session recordings, randomly sampled, with inter-rater reliability (e.g., intraclass correlation coefficient ≥.70) established on a calibration subset before ratings are used in outcome analyses; discrepancies above a pre-specified threshold should trigger a consensus rating procedure.

An important limitation of this framework, as currently specified, is that the MITI-based relational indicators capture motivational-interviewing-consistent behavior but not technical competence in delivering the CBT, schema-informed, or relapse-prevention components that make up the remainder of the protocol. Relational and technical competence are not interchangeable: a therapist could deliver an MI-consistent relational stance while conducting a technically inadequate functional analysis, or could deliver a technically correct cognitive restructuring exercise in a confrontational manner. Future fidelity work should therefore pair the MITI-based relational rating with a separate, PATH-G-specific technical competence checklist, rated on the same 1–7 anchored format, covering at minimum: the accuracy and specificity of ABC/functional analysis (whether antecedents, behavior, and consequences are each concretely identified rather than described in general terms); the adequacy of cognitive restructuring (whether the alternative thought generated is genuinely evidence-based rather than merely reassuring); the appropriateness of urge-delay and regulation technique selection to the patient’s presentation; and, where applicable, the coherence of schema-mode formulation with the material the patient has presented. This technical competence checklist has not yet been developed or piloted; its development is identified here as a priority for future fidelity research rather than presented as an existing instrument. These operational criteria, taken together, are proposed as a starting point for future feasibility studies and have not themselves been validated for PATH-G.

## 11 Therapist competencies, implementation requirements, and ethical boundaries

The workbook should be delivered only within structured psychotherapy and with clear clinical boundaries. It is designed for adults with GD, but the therapist must determine whether the patient can engage safely and meaningfully with workbook-based treatment. Severe psychiatric comorbidity, marked cognitive impairment, acute suicidality, manic states, psychosis, major intoxication, or profound social instability may require additional supports, slower pacing, adjunctive interventions, or alternative care pathways. This caution is supported by evidence on psychiatric comorbidity in problem and pathological gambling and by reviews emphasizing the need to advance integrated interventions for comorbid presentations (3, 51).

Recent NICE guidance is particularly relevant here. It recommends direct attention to suicidality in gambling-related harms, recognizes gambling as a potential dominant risk factor for suicidal ideation and attempts, and advises urgent referral when immediate risk is present (13). The protocol therefore treats safety planning as an essential implementation requirement rather than a peripheral administrative step. This is especially important because risk may intensify immediately after gambling episodes, during major losses, or following family disclosure.

Therapist competence is equally central. NICE recommends that the workforce delivering gambling treatment be trained and competent, and specifically notes that CBT should be delivered by psychologists or accredited CBT therapists (13). In the present model, competency involves more than technical knowledge of CBT. Therapists must also be able to manage ambivalence, shame, secrecy, dropout risk, and relapse processes in a non-stigmatizing, collaborative manner. They should be capable of using workbook content flexibly, modulating pace, tailoring literacy demands, protecting the therapeutic relationship, and integrating the workbook into a broader case formulation.

For the purposes of future feasibility studies, a minimum therapist training profile for PATH-G delivery is proposed: a recognized postgraduate qualification in clinical or counselling psychology, psychiatry, or psychotherapy; formal training in CBT to at least diploma level or equivalent; familiarity with motivational interviewing principles, verified by completion of a recognized MI training workshop (minimum 16 hours) or equivalent supervised practice; at least two years of post-qualification clinical experience, including direct work with addiction, behavioral disorders, or complex presentations involving shame, concealment, and relapse; and access to regular clinical supervision throughout protocol delivery, with a minimum of one supervision session per four treatment weeks. Schema-informed and experiential components in the later modules (Weeks 9–12 and maintenance) may be delivered with additional training in schema therapy or Gestalt-informed approaches, but this is not a prerequisite for core protocol delivery.

A clinically important implementation consideration concerns patients whose gambling is primarily or exclusively online. Online gambling disorder affects 15.8% of online casino/slot players and 8.9% of sports bettors, and practical barriers such as travel distance, financial and time costs, and stigma complicate access to structured therapies like CBT. The present protocol was designed with face-to-face delivery as the default modality, but several adaptations are required for online gamblers: trigger mapping in Week 3 must address digital rather than physical environmental cues (device proximity, app notifications, time-of-day routines, and social media exposure replace location-specific triggers); stimulus control strategies must include device-level interventions such as app blocking, self-exclusion from online platforms, and financial access restrictions; the observational exercise in Week 8 is contraindicated in its standard form and should be replaced by a guided observation of recorded online sessions or a structured reflection on the online gambling experience using written or visual prompts; because viewing recorded gambling sessions is itself a cue-exposure procedure (54), the same pre- and post-exercise craving ratings, contraindication criteria, and 48-hour check-in described above apply to this adaptation, with the additional safeguard that recorded material should be selected or pre-screened by the therapist to avoid unpredictable high-intensity stimuli such as near-miss sequences or large wins; between-session motivational anchors may be delivered via the same digital environment that maintains gambling risk, which requires clinical judgment about medium and timing. Internet-delivered CBT for gambling disorder has demonstrated feasibility in routine addiction care settings, suggesting that a blended or remote version of the present workbook is clinically plausible and warrants explicit attention in future implementation studies.

Clinical adaptation should also consider gender-specific presentations. Treatment-seeking women with gambling disorder show higher rates of anxiety and depression comorbidity compared to men, while men show higher rates of substance use problems; women also tend to have a later age of onset but faster progression of the disorder. Women with gambling disorder face greater overall psychiatric comorbidity and are more likely to present with mood disorders, suicidality, mania, anxiety, and alcohol dependence. These differences suggest, as a clinical hypothesis rather than an evidence-based recommendation, that protocol delivery for women may benefit from greater emphasis on emotion regulation modules (Weeks 5–6), more careful safety monitoring, and earlier introduction of schema-informed work addressing shame and self-punitive patterns; no direct trial evidence yet supports these specific adaptations, and they are proposed here as testable modifications for future research rather than established practice. In women, dropout risk from CBT for gambling disorder is higher among those with lower GD severity and higher psychopathological distress, precisely the profile that may benefit most from the intensive motivational work embedded throughout this protocol. Future implementation studies should therefore analyze outcomes by gender as a primary moderator rather than a demographic covariate.

The protocol may be adaptable to outpatient psychotherapy, community gambling services, and blended care settings, but such adaptation should preserve several non-negotiable elements: therapist oversight, motivational review at transition points, risk-sensitive use of homework, a clear maintenance phase, and a structured relapse framework. Without these components, the workbook could easily be reduced to an over-prescriptive self-monitoring package detached from therapeutic containment.

A final implementation consideration concerns therapist variability. Protocol manuals reduce but do not eliminate therapist effects on outcome. The therapeutic stance described throughout this protocol (empathic, non-stigmatizing, collaborative, ownership-promoting) is not merely a stylistic recommendation; broader psychotherapy research indicates that relational and alliance-related factors are consistently associated with outcome across treatment modalities (50), and we propose, as a testable hypothesis rather than an established finding, that this stance may function as an additional mechanism of change in GD specifically, given the salience of shame, concealment, and demoralization as barriers to engagement in this population (6). Therapists who deliver the workbook with high fidelity to its technical content but low fidelity to its relational stance may, on this hypothesis, achieve more limited motivational and identity-related gains than those targeted in the later phases of the protocol; this remains to be tested empirically. Future implementation and fidelity research should therefore assess both adherence (delivery of prescribed techniques) and competence (quality of MI-consistent relational delivery) as distinct dimensions, consistent with established frameworks for evaluating fidelity in complex psychological interventions.

## 12 Clinical strengths and limitations

Several strengths of the protocol stand out. First, it is highly operationalized without becoming mechanically narrow. Therapists are given a sequence and a set of tools, while still retaining responsibility for case formulation and individualization. Second, the model links symptom-focused work to emotional, motivational, and identity-level processes, which is especially valuable in a disorder where gambling often functions as both behavior and psychic regulator. Third, the protocol explicitly honors continuity of care: motivation is revisited, maintenance is formalized, and relapse is anticipated rather than treated as treatment failure. Fourth, the workbook materials are unusually rich in between-session devices, which may improve ecological validity by placing change where it actually occurs, in daily life.

The protocol also has limitations. Most importantly, the present protocol overlaps substantially with Cognitive Motivational Behavior Therapy (CMBT; 29–31), which remains the closest, and best-evidenced, integrative framework for gambling disorder. Any future trial of this protocol should build in an active comparator arm, or should specify in advance the patient profiles, clinical contexts, or treatment phases in which we would expect the additional components to add something CMBT does not already provide.

A second limitation concerns evidentiary asymmetry across components. CBT and motivational work have stronger direct relevance to gambling treatment than some of the workbook’s experiential, schema-informed, or humanistic elements. PATH-G addresses this by explicitly positioning those latter components as adjunctive and formulation-driven, but future studies should test whether they add incremental value to core treatment.

A third limitation is implementation complexity. The protocol asks a great deal of both therapist and patient: repeated homework, emotional reflection, motivational review, behavioral experiments, and later identity work. This richness is clinically attractive, but it may also increase burden and dropout risk for some patients. Future research should therefore examine feasibility, treatment adherence, attrition, therapist fidelity, and which patient profiles benefit most from the full model versus a simplified version. This limitation should be operationalized through explicit implementation outcomes rather than treated as a purely narrative concern (58).

A fourth limitation is that some later-phase exercises, especially phenomenological work with emptiness, values, and identity, are harder to standardize for multisite research. However, this difficulty may also reflect a clinical truth: long-term recovery from GD often involves precisely these dimensions. The challenge for future protocol refinement is to preserve depth while improving reproducibility.

A fifth limitation is the absence of explicit hypotheses regarding moderators of differential treatment response. The protocol is designed as a universal pathway, but it is clinically plausible that different patient profiles derive differential benefit from different components. Specifically, patients with high readiness to change may derive relatively less incremental benefit from the intensive motivational work in early modules, while patients with low baseline readiness, precisely the group where CMBT has shown differential advantage over standard CBT (31), may constitute the primary target population for the protocol’s motivational architecture. Conversely, patients with marked schema-level vulnerabilities (chronic emptiness, punitive self-representations, early maladaptive schemas) may benefit most from the third-layer components introduced in Weeks 9–12 and maintenance. Future research should specify these moderator hypotheses *a priori* rather than treating the protocol as uniformly applicable across the full clinical range.

## 13 Conclusion

This manuscript presents PATH-G (Progressive Adaptive Treatment Hierarchy for Gambling Disorder), a 12-week workbook-assisted psychotherapy protocol that offers a clinically rich and methodologically explicit model for the treatment of Gambling Disorder. The central contribution of PATH-G is not the invention of a new school of therapy, but the disciplined integration of multiple treatment functions into a single progressive pathway: CBT and motivational work as the core; mindfulness and urge-regulation strategies as adjunctive supports; schema-informed, reflective, and experiential methods as depth-oriented tools for maintenance and identity reconstruction.

The manuscript also clarifies a point that is often underdeveloped in protocol descriptions: motivation is not merely something assessed at intake but a therapeutic process to be supported across the whole course of treatment. By making motivational work explicit, through stage-sensitive assessment, collaborative goal revision, discrepancy work, values clarification, recommitment after lapses, and an empathic non-stigmatizing stance, PATH-G becomes more coherent with both clinical reality and current guidance. Cognitive Motivational Behavior Therapy (CMBT; 29–31) is the best-evidenced precedent for combining CBT and MI in this way, and PATH-G is conceptually indebted to it. PATH-G extends this precedent in three directions: turning between-session change into a structured, week-by-week workbook process rather than an assumed byproduct of sessions; building out a formal maintenance phase that works on identity reconstruction, values-based living, and tolerance of experiential absence; and adding schema-informed and experiential work in the later phases to reach the emotional layers underneath chronic gambling. Whether these extensions provide added value beyond what CMBT already delivers remains an open empirical question. Once feasibility and outcome data support the protocol, a head-to-head comparison with CMBT would be a useful next step, with the hypotheses about which patients, phases, or outcomes should differ specified in advance.

In summary, PATH-G shares CMBT’s core logic that motivational work should accompany CBT throughout treatment rather than being confined to its outset, and extends this logic with an operationalized workbook, a formal maintenance phase, and later-stage schema-informed and experiential work. Whether this extension yields outcomes beyond those already produced by CMBT remains a question for future research, ideally through a head-to-head trial with CMBT as the active comparator.
