# Supplementary material for: PATH-G: a proposed integrative motivational-cognitive-behavioral protocol for gambling disorder. A theoretically grounded 12-week workbook-based model with maintenance and relapse components
Source: Front Psychiatry. 2026 Sep 10;17:1871785. doi: 10.3389/fpsyt.2026.1871785 (PMC13601271; doi:10.3389/fpsyt.2026.1871785)
Supplement: Supplementary file 1 [file DataSheet1.pdf]

Appendix A. Illustrative Session Outlines

The following outlines illustrate the structure of three representative PATH-G sessions: Session 1 (engagement and assessment), Session 5 (urge management and emotional regulation), and Session 11 (values clarification and identity consolidation). These outlines are not prescriptive scripts. Clinical judgment governs pacing, emphasis, and adaptation to individual patient profiles, risk level, and session context. The "Key therapist stance" column reflects the relational and motivational quality of delivery, which is considered a mechanism of change in its own right, not merely a stylistic preference.

A.1 Session 1 — Engagement, Assessment, and Collaborative Commitment

Estimated duration: 75–90 minutes. Sessions 1 and 6 are the two sessions designated for extended time to accommodate comprehensive assessment and mid-treatment review respectively.

Phase	Content	Time	Key therapist stance
Opening and orientation	Introduce the PATH-G protocol structure, the workbook rationale, and the collaborative therapeutic stance. Establish the distinction between the in-session work and the between-session workbook tasks. Explain confidentiality and its limits.	10 min	Warm, non-stigmatising; normalise the decision to seek help.
Baseline assessment	Gambling history (type, duration, escalation); functional mapping of the role gambling plays; initial motivational scaling: importance (0–10), confidence (0–10), readiness (0–10).	25 min	Curious and collaborative; treat data as estimates, not confessions.
Psychoeducation	Brief introduction to the cognitive-behavioural cycle of GD: triggers → urges → cognitive distortions → gambling behaviour → consequences → new triggers. Normalise ambivalence explicitly.	15 min	Didactic but dialogical; check understanding; invite questions.
Collaborative Treatment Commitment and Safety Plan	Jointly draft: patient's stated reasons for change; initial goals; identified risks (suicidality, financial urgency, online access); agreed safety contacts and protective measures.	15 min	Ownership-promoting; the plan belongs to the patient, not to the therapist.
Between-session task assignment	Daily gambling diary (time, money, urges, emotions); initial trigger log. Explain the purpose: not surveillance, but making the invisible visible.	5 min	Frame homework as clinical tool, not compliance test.
Closing	Summarise session content; elicit patient's reaction; confirm next appointment; note any immediate safety concerns.	5 min	Reassuring; leave space for questions or disclosures.

Note. The Collaborative Treatment Commitment and Safety Plan drafted in Session 1 is a living document: it is reviewed at Session 6 (mid-treatment), after any lapse, and at the transition to the maintenance phase. Suicidality, financial urgency, and access to gambling platforms are assessed at this session and revisited at every subsequent session as clinical indicators require.

A.2 Session 5 — Urge Management and Emotional Regulation

Estimated duration: 50 minutes. Standard session length for Sessions 2–12, except Session 6.

Phase	Content	Time	Key therapist stance
Check-in and homework review	Review gambling diary and trigger log from Weeks 3–4. Identify patterns; reinforce monitoring effort; note any lapses without shaming.	10 min	Validating; reinforce self-observation as achievement.
Urge delay — urge surfing	Patient describes a recent urge in detail while therapist guides attention to physical sensations, emotional tone, and the natural arc of urge intensity over time. Demonstrate that urges peak and subside without action.	15 min	Grounded, present-focused; tolerate silence; normalise physical sensations.
Emotion regulation	Identify the primary emotional antecedent to the reported urge (boredom, shame, anger, loneliness, excitement). Introduce and practise one regulation strategy suited to the patient's profile: slow breathing, grounding 5-4-3-2-1, urge surfing, or a self-compassionate statement.	15 min	Experiential, not only verbal; practise in session, not just explain.
Graded substitution review	Review the substitution plan from Week 4. Reinforce visible gains. Expand the non-gambling activity schedule for Week 5, with at least one activity pre-scheduled in a high-risk time slot.	7 min	Concrete and specific; ask what the activity actually felt like.
Between-session task	Urge delay practice log; one additional non-gambling activity pre-scheduled in writing for a high-risk moment.	3 min	Brief and clear; check patient has noted the task in the workbook.

Note. Regulation techniques introduced in Session 5 are practised in session before being assigned as between-session tasks. Where urge surfing or grounding are new to the patient, the therapist demonstrates the technique first and guides the patient through it with the patient's own recent urge as material. Techniques presented only verbally are less likely to be accessed during high-intensity craving states.

A.3 Session 11 — Values Clarification and Identity Consolidation

Estimated duration: 50 minutes. Sessions 10–12 form the consolidation and identity phase of PATH-G, shifting the clinical focus from urge management to the construction of a sustainable non-gambling life structure.

Phase	Content	Time	Key therapist stance
Check-in	Brief review of week, mood, urge frequency, and workbook completion. Note any high-risk episodes since last session.	8 min	Attentive to tone; notice signs of fatigue or demoralization.
Values clarification	Patient selects 3–5 personal values from the workbook list. For each, the therapist elicits one concrete present-tense action the patient already performs or can commit to this week. Frame explicitly: not "Who do I wish to become?" but "How do I live this value today?"	20 min	Evocative and grounded; connect values to specific behaviours, not abstractions.
Identity dialogue	Brief structured dialogue — written or spoken — between the patient's Healthy Adult voice and the part still drawn to	12 min	Non-judgmental; validate both

Phase	Content	Time	Key therapist stance
	gambling. Therapist facilitates differentiation of these positions without pathologising either.		voices before helping the Healthy Adult strengthen.
Motivational consolidation	Revisit the original reasons for change from the Collaborative Treatment Commitment. Identify what has shifted since Week 1. Name visible gains explicitly.	5 min	Reinforcing; help the patient own the progress, not attribute it to luck.
Between-session task	Values diary: one brief daily entry noting a moment where a chosen value was enacted, however small. Can be written, audio-recorded, or noted in a simple table.	5 min	Low-demand format; adapt to literacy level if needed.

Note. The identity dialogue in Session 11 is not a structured schema mode work session; it is a briefer, more conversational version of the same principle. Full schema-informed mode dialogue is introduced in Sessions 9–10. By Session 11, patients who have engaged with the earlier work will typically be familiar with the distinction between the Healthy Adult and the Impulsive or Vulnerable Child voices and can use that vocabulary to structure the dialogue.

Appendix B. Examples of Key Workbook Exercises

The following exercises are presented in their operational format, including the structured fields and reflective prompts that distinguish them from narrative description. They are drawn directly from the PATH-G workbook. All exercises are introduced and explained by the therapist before being assigned as between-session tasks; none are designed for unsupported self-administration.

B.1 Weekly Gambling and Urge Diary (Weeks 1–8)

Complete one row per day. Circle or underline emotions where a list is preferred. If no gambling occurred, complete the urge and emotion columns regardless: monitoring absence is as important as monitoring occurrence.

Day	Gambled? (Y/N)	Time spent	Money spent/lost	Urge (0–10)	Emotion before	Emotion after	Situation / trigger
**Monday**							
**Tuesday**							
**Wednesday**							
**Thursday**							
**Friday**							
**Saturday**							
**Sunday**							

End-of-week reflection (complete on Sunday or the morning of your next session):

What pattern do I notice across the week?

Which day or moment was hardest? What was happening?

What am I most proud of from this week, however small?

What do I want to work on differently next week?

Note. The diary is not a report card. Its purpose is to make the automatic visible. Incomplete days or entries with missing data are preferable to no diary at all. The therapist reviews the diary collaboratively, not as an evaluator.

B.2 Graded Substitution Planner (Weeks 2–10)

For each gambling occasion you plan to replace this week, identify a specific substitute activity in advance. The activity does not need to be enjoyable; it needs to be real, concrete, and scheduled before the high-risk moment arrives.

Gambling occasion to be replaced	Substitute activity	Time & date	With whom (if anyone)	How it went (complete after)

Reflection (complete at the end of the week):

What did I notice about the substitute activity compared to gambling?

What made it harder or easier than expected?

Did I follow through? If not, what got in the way?

What would I do differently next week?

Note. Substitution is not distraction. It is the active construction of a daily ecology in which gambling becomes progressively less necessary and less available. The "how it went" column is as important as the plan itself: it provides the raw material for collaborative review in the following session.

B.3 Post-Lapse Recovery Plan (Relapse Module)

Complete this form within 24–48 hours of a lapse. Do not wait until you feel ready. The purpose is not to punish yourself but to understand what happened and to stabilise the situation before it escalates. Work through the prompts in order.

Prompt	My response (complete within 24–48 hours of lapse)
**What happened?**	(Describe briefly and without self-judgment: when, where, how long, how much)
**Emotional state immediately before the lapse**	(e.g., boredom, shame, anger, loneliness, excitement, financial pressure)

Prompt	My response (complete within 24–48 hours of lapse)
**Schema mode that became dominant**	(e.g., Impulsive Child, Vulnerable Child, Detached Protector, Punitive Critic)
**What would my Healthy Adult say about this episode?**	
**Who will I contact in the next 24 hours?**	(Name and contact:)
**One action I can take today to stabilise the situation**	
**Financial protection step I will take now**	(e.g., transfer money out of reach, delete app, call self-exclusion service)
**What does recommitting to PATH-G look like this week?**	(One specific, concrete step:)
**Message from my Healthy Adult to myself right now**	

Note. A lapse is not a relapse. A lapse is a single episode or brief return to gambling. A relapse is a sustained return to the previous pattern. PATH-G distinguishes between the two because the appropriate clinical response differs: a lapse calls for rapid stabilisation and recommitment; a full relapse may require intensification of clinical contact, revision of the protection plan, and review of the maintenance phase. The post-lapse plan is activated by the patient independently and then reviewed with the therapist at the next scheduled session or sooner if clinically indicated.

Note on adaptation. All three exercises can be adapted for patients with low literacy (oral completion with therapist scribing; simplified visual formats; audio-recorded reflections) or for online gambling presentations (urge diary adapted to device and time-of-day rather than location). See Section 11 of the main manuscript for implementation guidance.
